# Evidence for a causal link between adaptor protein PDZK1 downregulation and Na^+^/H^+^ exchanger NHE3 dysfunction in human and murine colitis

**DOI:** 10.1007/s00424-014-1608-x

**Published:** 2014-10-02

**Authors:** Sunil Yeruva, Giriprakash Chodisetti, Min Luo, Mingmin Chen, Ayhan Cinar, Lisa Ludolph, Maria Lünnemann, Julia Goldstein, Anurag Kumar Singh, Brigitte Riederer, Oliver Bachmann, Andre Bleich, Markus Gereke, Dunja Bruder, Susan Hagen, Peijian He, Chris Yun, Ursula Seidler

**Affiliations:** 1 Department of Gastroenterology, Hepatology and Endocrinology, Hannover Medical School, Carl-Neuberg-Straße 1, 30625 Hannover, Germany; 2 Institute for Animal Research, Hannover Medical School, Hannover, Germany; 3 Immune Regulation Group, Helmholtz Centre for Infection Research, Braunschweig, Germany; 4 Department of Surgery, Beth Israel Deaconess Medical Centre, Boston, USA; 5 Division of Digestive Diseases, Department of Medicine, Emory University, Atlanta, USA; 6 Infection Immunology Group, Department of Medical Microbiology, Otto von Guericke University Magdeburg, Magdeburg, Germany; 7 Present Address: The College of Medicine, Shaoxing University, No. 900 Chengnan Avenue, Shaoxing, 312000 Zhejiang Province China; 8 Present Address: Ministry of Health, Provincial health directorate of Istanbul, Istanbul, Republic of Turkey

**Keywords:** pH regulation, Sodium absorption, Electrolyte transport, Intestinal inflammation, Inflammatory bowel disease

## Abstract

A dysfunction of the Na^+^/H^+^ exchanger isoform 3 (NHE3) significantly contributes to the reduced salt absorptive capacity of the inflamed intestine. We previously reported a strong decrease in the NHERF family member PDZK1 (NHERF3), which binds to NHE3 and regulates its function in a mouse model of colitis. The present study investigates whether a causal relationship exists between the decreased PDZK1 expression and the NHE3 dysfunction in human and murine intestinal inflammation. Biopsies from the colon of patients with ulcerative colitis, murine inflamed ileal and colonic mucosa, NHE3-transfected Caco-2BBe colonic cells with short hairpin RNA (shRNA) knockdown of PDZK1, and Pdzk1-gene-deleted mice were studied. PDZK1 mRNA and protein expression was strongly decreased in inflamed human and murine intestinal tissue as compared to inactive disease or control tissue, whereas that of NHE3 or NHERF1 was not. Inflamed human and murine intestinal tissues displayed correct brush border localization of NHE3 but reduced acid-activated NHE3 transport activity. A similar NHE3 transport defect was observed when PDZK1 protein content was decreased by shRNA knockdown in Caco-2BBe cells or when enterocyte PDZK1 protein content was decreased to similar levels as found in inflamed mucosa by heterozygote breeding of Pdzk1-gene-deleted and WT mice. We conclude that a decrease in PDZK1 expression, whether induced by inflammation, shRNA-mediated knockdown, or heterozygous breeding, is associated with a decreased NHE3 transport rate in human and murine enterocytes. We therefore hypothesize that inflammation-induced loss of PDZK1 expression may contribute to the NHE3 dysfunction observed in the inflamed intestine.

**Electronic supplementary material:**

The online version of this article (doi:10.1007/s00424-014-1608-x) contains supplementary material, which is available to authorized users.

## Introduction

One of the most common symptoms of patients with inflammatory bowel disease (IBD) is diarrhea [33]. Previous observations by us and others suggested that intestinal inflammation may result in a decrease of mucosal fluid absorption via dysregulated sodium transport [10, 13, 26, 28, 34, 40]. Since the Na^+^/H^+^ exchanger isoform NHE3 is strongly expressed in the surface cells of the colon of humans and rodents, this transporter was studied as one potential candidate of mediating the reduction in intestinal Na^+^ absorption in inflammatory conditions. However, changes in NHE3 expression levels in human IBD or in murine colitis models were not a consistent finding [13, 24, 26, 34, 40], and its apical membrane localization was found to be preserved in murine and human colitis [24, 40]. However, in all studies where NHE3 function was assessed in the inflamed intestine, it was found to be defective [4, 13, 24, 40]. We and others therefore considered the possibility of a defect in the regulation of NHE3 transport activity caused by changes in expression of one or several of its interacting partners [13, 24, 26, 40].

NHE3 transport function is regulated in multiprotein complexes [1, 11, 12, 17]. The NHERF family of PDZ proteins binds to NHE3 as well as other components of these complexes and thereby regulates NHE3 membrane mobility as well as its interaction with signaling molecules [11, 22]. Genetic deletion of each of the NHERF proteins interferes with the regulation of NHE3 by second messengers in cellular expression systems [32, 41] or in murine intestine [7–9]. In the PDZK1-deficient as well as the NHERF1-deficient, but not in the NHERF2-deficient mouse, intestinal Na^+^ absorption as well as acid-activated NHE3 activity are decreased [7–9, 19].

In search for defects in the NHE3 regulatory system in inflamed intestine, Sullivan et al. found decreased protein expression of both NHE3 as well as NHERF1 and NHERF2 in colonic biopsies from IBD patients [34]. We observed a strong downregulation of PDZK1 (NHERF3) but not of NHERF1 and NHERF2 messenger RNA (mRNA) and protein in a mouse model of chronic colitis [24]. In the present study, we therefore focused on the role of PDZK1 in inflammatory NHE3 dysfunction and addressed the following questions: (a) Is the downregulation of PDZK1 a general phenomenon associated with intestinal inflammation in murine and human intestine? and (b) Is PDZK1 downregulation associated with NHE3 dysfunction, irrespective of its cause, in both murine and human NHE3 expressing enterocytes?

## Material and methods

### Reagents and antibodies

Unless stated otherwise, all chemical reagents were purchased from either Applichem (Darmstadt, Germany) or Sigma-Aldrich (Munich, Germany), and all cell culture reagents were purchased from either PAA laboratories (Cölbe, Germany) or Biochrom AG (Berlin, Germany). Rabbit polyclonal PDZK1 antibody (PAB15564) was from Abnova (Jhongli City, Taoyuan County, Taiwan), rabbit polyclonal NHE3 antibody (NHE31-A) was from Alpha diagnostic (San Antonio, USA), and rabbit polyclonal β-actin antibody (ab8227) was from Abcam (Cambridge, UK).

### Mice breeding

All animal experiments were performed according to national and institutional guidelines and were approved by the Hannover Medical School Committee on investigations involving animals as well as by an independent committee of the regulatory agency (Niedersächsisches Landesamt für Verbraucherschutz). Three mouse models were studied: The *Tnf* ^ΔARE+/−^ mice (and wild type (WT) controls) develop an ileocolitis, as described previously [20]. The 2 % dextran sodium sulfate (DSS)-induced *Il-10* ^−/−^ mice develop a moderately severe colitis (this paper). The *Rag2* ^−/−^ CD4^+^ CD45^RBhigh^ transfer colitis mice (described in Supplementary methods) develop colitis with more associated systemic disease and diarrhea than the other mouse models. *Pdzk1* ^−/−^ mice on the Sv129 background have previously been described [9, 19].

### Patient selection

Informed consent was obtained prior to endoscopy/surgery. For the control group, we selected patients with a healthy digestive tract (neither macroscopic nor microscopic changes). The protocols were approved by the Hannover Medical School Ethics committee. The characteristics of both healthy control patients and ulcerative colitis (UC) patients are given in the Supplementary Table 1 and 2. Histological scoring of inflammation status of the biopsies were performed as described [13, 40]. The biopsies were graded retrospectively based on histological score, not by cytokine levels or endoscopic score. If there were discrepancies, or if the medical history of the patients was not completely available, the biopsy was not used. From all biopsies taken, we eventually selected those taken from the sigmoid colon only, because this corresponded to the site from which the biopsies were taken for functional NHE3 activity measurements in human colonocytes [13, 40]. Because many biopsies had to be discarded, we pooled all biopsies with the inflammatory grades mild, moderate, and severe for the present analysis.

### Light and electron microscopy

For *Il-10* ^−/−^ mice, processing of the tissues and histological score determination was done as described previously [6], and an example of the morphology is shown in the Supplementary Figure 1. *Tnf* ^ΔARE+/−^ intestinal histology was described previously [39]. For electron microscopy, intestinal sections were fixed and imaged according to previously described protocols [15].

### Cell culture and lentiviral-mediated PDZK1 knockdown in Caco-2BBe/hNHE3V cells

The generation of Caco-2BBe/hNHE3V cells stably expressing human NHE3 tagged with vesicular stomatitis virus protein G (VSV-G) epitope at the C-terminus was performed according to a method described previously by Lin et al. [25]. Lentiviral particles were generated as described in [18]. For the details of PDZK1 knock down Supplementary files. Knockdown was stable for up to 25 passages (not tested for longer time periods), and all the experiments were done within these passages. Acid suicide selection to maintain NHE3 expression levels in the Caco-2BBe/hNHE3V cells was done as previously described [37], with modifications as described in the Supplementary files.

### RNA isolation and real-time PCR

RNA isolation and quantitative rtPCR was done according to previously detailed methods [19], with modifications described in the Supplementary files, where a list of primers is given.

### Immunohistochemistry and confocal microscopy

Mice were sacrificed by cervical dislocation and tissues were washed with PBS, fixed with 2 % paraformaldehyde at 4 °C for 2 h, followed by overnight incubation with 30 % sucrose in PBS. Immunohistochemistry and confocal microscopy for NHE3 were done as described previously [25]. NHE3 distribution in the brush border membrane (BBM) was studied as described previously [8].

### NHE3 activity measurements in intact villi and isolated colonic surface enterocytes

The method for pH_i_ measurement and assessment of acid-activated NHE3 activity in intact microdissected murine ileal villi and isolated murine colonic crypts has been previously described [3, 8, 9].

pH_i_ fluorometry was performed in Caco-2BBe/hNHE3V cells as described previously [25], with modifications. Briefly, cells grown on 25-mm round cover slips were loaded with 5 μM BCECF-AM (Invitrogen, Darmstadt, Germany) in solution A for 30 min at 22 °C, mounted in a custom-made perfusion chamber, perfused with oxygenated buffer A (see the Supplementary Table 4 for buffer composition) for 20 min, followed by an ammonium prepulse (32 mM NH_4_Cl replaced 32 mM NaCl), followed by solution B. Solution A was used in the pH_i_ recovery phase. 50 μM of HOE642 was maintained in all the experiments during acidification and recovery phases. Experiments were calibrated with 10 μM nigericin in solution D. Acid-activated NHE3 activity was measured by calculating the slope of the initial 15 s during recovery phase (dpH_i_/dt), and multiplying this value by the buffering capacity of the cell at a particular pH_i_ during recovery to measure the H^+^ efflux by NHE3.

### Brush border membrane (BBM) preparation and immunoblot assay

Isolation of brush border membrane from the small intestine of *Pdzk1* ^+/+^, ^+/−^, and ^−/−^ mice and Western immunoblot assays were performed as described previously [19]. Total tissue or scraped mucosa lysates were prepared as described previously [20]. 50 μg protein was loaded on to the SDS-PAGE gel and probed with anti-PDZK1 antibody. Further details are given in the Supplementary files.

### Statistical analysis

Results are given as means ± SEM. Student’s *t* test with Welch’s correction was used for comparing between two groups. One-way or two-way ANOVA with post hoc analysis was used for multiple comparisons.

## Results

### NHE3, NHERF1, PDZK1, and inflammatory cytokine gene expression in sigmoid colonic biopsies

**Fig. 1:**
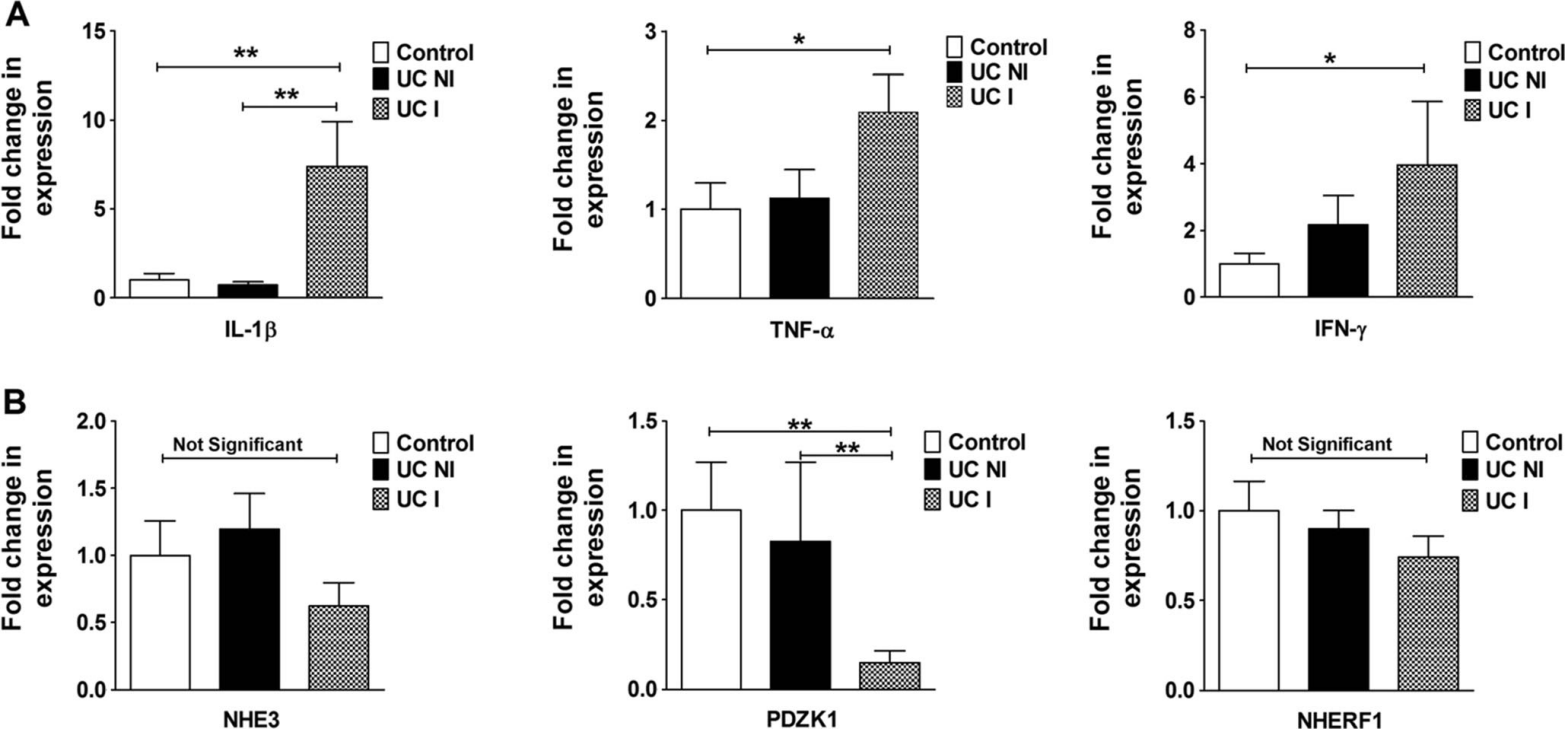
Gene expression in the inflamed human intestine. **a** IL-1β, TNF-α, and IFN-γ mRNA expression and **b** NHE3, PDZK1, and NHERF1 mRNA expression, in the sigmoid colon biopsies of control individuals (no colonic inflammation), ulcerative colitis noninflamed (UC NI), and ulcerative colitis inflamed (UC I) patients. mRNA expression levels of all three proinflammatory cytokines were significantly increased in UC I vs UC NI and controls (A). PDZK1 mRNA expression was significantly decreased in inflamed colonic mucosal biopsies from UC patients (UC I) compared to biopsies taken from noninflamed controls, and from UC patients without active inflammation (UC NI), from the same colonic segment. *Bar graphs* are represented as ± SEM.*P < 0.05, **P < 0.005, ***P < 0.0005. *n* = controls (16), UC NI (13), UC I (14). A geometrical mean of three housekeeping genes were used as the reference gene [29, 38]

IL-1β, TNF-α, and INF-γ mRNA levels were unaltered between control and noninflamed UC patients but were significantly elevated in inflamed UC biopsies (Fig. 1a). A strong downregulation of PDZK1 expression was seen in inflamed UC mucosa but not in noninflamed UC mucosa and healthy controls, while both NHE3 and NHERF1 mRNA expression levels were not significantly different between the groups (Fig. 1b).

### TNF-α, IL-1β, NHE3, NHERF1, and PDZK1 gene expression in murine models of intestinal inflammation

**Fig. 2:**
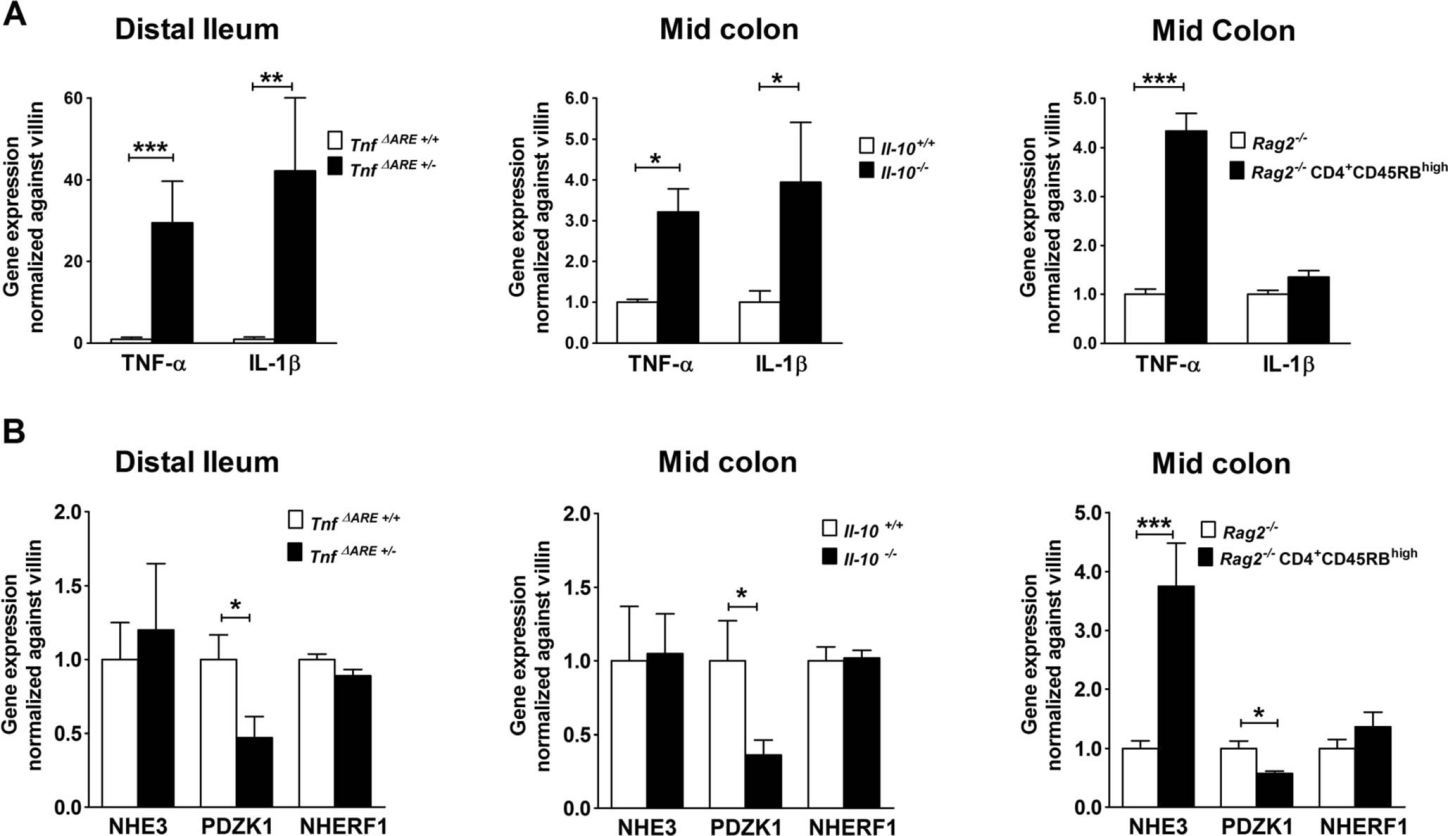
Gene expression in the inflamed murine intestine. **a** TNF-α and IL-1β mRNA expression and **b** NHE3, PDZK1, and NHERF1 mRNA expression in distal ileum or mid-colon in the three mouse models as described in the text. **a** The proinflammatory cytokines were elevated in the inflamed vs control intestinal mucosa, but the pattern of cytokine increase was different in the different models both qualitatively and quantitatively. **b** Nevertheless, a significant decrease in PDZK1 mRNA expression was observed in the inflamed distal ileum of *Tnf* ^ΔARE+/−^ and mid-colon of *Il-10* ^−/−^ and *Rag2* ^−/−^ CD4^+^ CD45^RBhigh^ mice compared to their respective controls. NHE3 mRNA was increased in the fairly acute *Rag2* ^−/−^ CD4^+^ CD45^RBhigh^ transfer colitis mouse model. *Bar graphs* are represented as mean ± SEM. *n* = 5–6 pairs of mice. *P < 0.05, **P < 0.005, ***P < 0.0005

TNF-α mRNA expression levels were found to be elevated in all three mice models, whereas IL-1β mRNA expression was only elevated in *Tnf* ^ΔARE+/−^ and *Il-10* ^−/−^ mice models compared to noninflamed littermates (Fig. 2a). Despite the strong variations in the cytokine expression levels, PDZK1 mRNA expression was significantly decreased compared to controls in the intestine of all inflamed mouse models (Fig. 2b). NHE3 and NHERF1 mRNA expressions were unaltered in the distal ileum of *Tnf* ^ΔARE+/−^ mice and the colon of *Il-10* ^−/−^ mice compared to their respective controls, and NHE3 mRNA was even increased in *Rag2* ^−/−^ CD4^+^ CD45^Rbhigh^ mice (Fig. 2b). The relationship of PDZK1 to NHE3 mRNA expression was thus markedly decreased in all inflamed epithelia compared to the noninflamed controls.

**Fig. 3:**
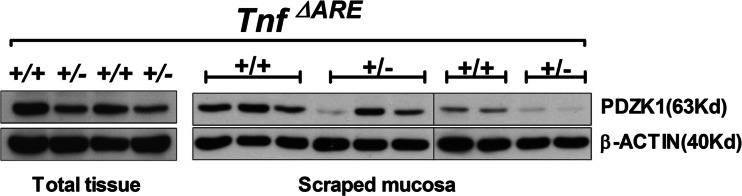
PDZK1 protein expression in the distal ileum of *Tnf* ^ΔARE+/+^ and ^+/−^ mice. PDZK1 protein expression was analyzed by Western analysis in the total tissue lysates (*n* = 2 pairs of mice) and scraped mucosa (*n* = 5 pairs of mice) of distal ileum from *Tnf* ^ΔARE^ mice. PDZK1 protein abundance was found to be strongly decreased in both scraped mucosa and total tissue lysates from the distal ileum of *Tnf* ^ΔARE+/−^ mice as compared to WT littermates

Protein expression of PDZK1 was measured in total tissue lysates or in scraped mucosa of the distal ileum in *Tnf* ^ΔARE+/+^ and *Tnf* ^ΔARE+/−^ mice, the strong reduction in PDZK1 protein content was confirmed both in total tissue lysates and in the scraped mucosa (Fig. 3).

### NHE3 localization in the BBM of inflamed and noninflamed regions of the intestinal tract of murine models of ileitis and colitis

**Fig. 4:**
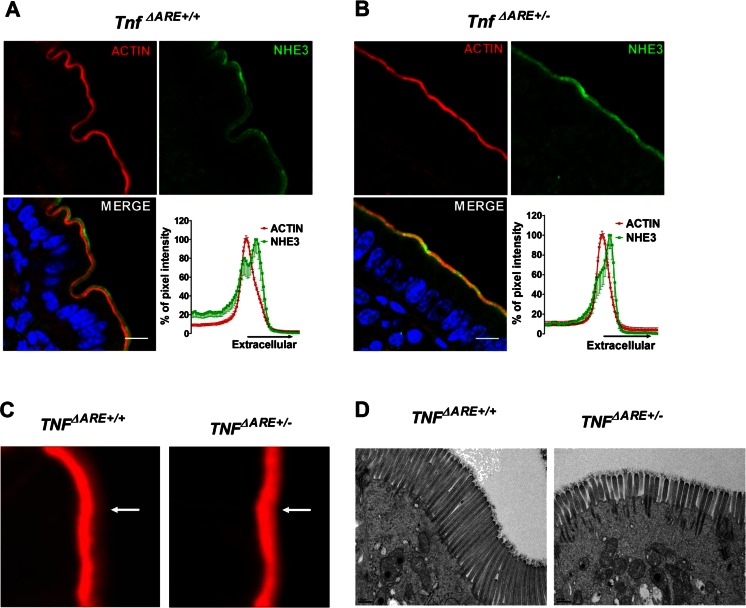
NHE3 localization and microvillar length in the distal ileum of *Tnf* ^ΔARE+/+^ and ^+/−^ mice. **a** In the distal ileum of *Tnf* ^ΔARE+/+^ mice, NHE3 was found both in the intervillar cleft/terminal web region (the NHE3 which colocalizes with the peak of the F-actin signal) and in the microvillar region (the peak that is more extracellular to the peak of the F-actin signal). **b** In the BBM of *Tnf* ^ΔARE+/−^ mice, overall NHE3 immunofluorescence was not decreased compared with noninflamed littermates, but the peak of NHE3 was closer to that of the F-actin signal compared to the WT controls. Images are representative of three individual experiments done in at least three pairs of mice. *Scale bar* represents 10 μM. **c** High magnification of F-actin staining in the BBM revealed that the microvillar staining (hazy red zone pointed to with *white arrowhead*) was decreased in the distal ileum *Tnf* ^ΔARE+/−^ compared to *Tnf* ^ΔARE+/+^. Figure represents data observed in three mice pairs. **d** Electron microscopy pictures taken from *Tnf* ^ΔARE+/+^ and *Tnf* ^ΔARE+/−^ mice distal ileum showing reduced microvilli lengths in the *Tnf* ^ΔARE+/−^ mice; data represents the results from two pairs of mice. *Scale bar* represents 0.5 μM

In chronically inflamed distal ileum of the *Tnf* ^ΔARE+/−^ mouse, the NHE3 staining in relation to that of F-actin, which outlines the microvilli and the terminal web region, showed less spatial separation between the maximal intensity of F-actin (terminal web region) and NHE3 (Fig. 4a, b). F-actin staining revealed a reduction of the hazy red zone at the luminal side of the BBM in the *Tnf* ^ΔARE+/−^ ileum compared to its noninflamed littermates indicative of shorter microvilli (Fig. 4c). Electron microscopical investigations confirmed the shortening of the microvilli in the chronically inflamed ileum of *Tnf* ^ΔARE+/−^ mice (Fig. 4d).

**Fig. 5:**
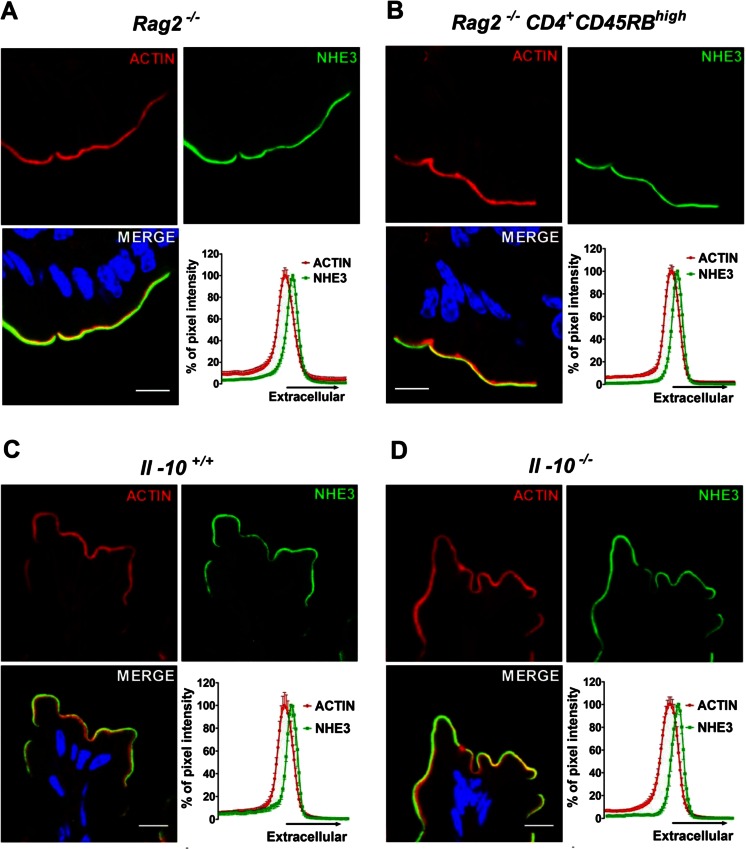
NHE3 localization in the intestinal BBM of murine models of colitis. No change in NHE3 localization in the BBM of **a** *Rag2* ^−/−^ vs **b** *Rag2* ^−/−^ CD4^+^ CD45^RBhigh^ and **c** *Il-10* ^+/+^ vs **d** *Il-10* ^−/−^ mice colon was found. Images are representative of three individual experiments done in at least three pairs of mice. *Scale bar* represents 10 μM

Confocal imaging did not reveal any alteration in the intensity or relative distribution of NHE3 protein in relation to F-actin, in the *Rag2* ^−/−^ CD4^+^ CD45^RBhigh^ vs *Rag2* ^−/−^ colon (Fig. 5a, b), as well as in the colon of *Il-10* ^+/+^ and ^−/−^ mice (Fig. 5c, d). Electron microscopy was not performed for colonic tissue. Taken together, despite shorter microvilli in inflamed ileum, the brush border membrane localization of NHE3 was preserved in inflamed intestinal mucosa of all three mouse models.

### NHE3 transport activity in inflamed ileal or colonic enterocytes

**Fig. 6:**
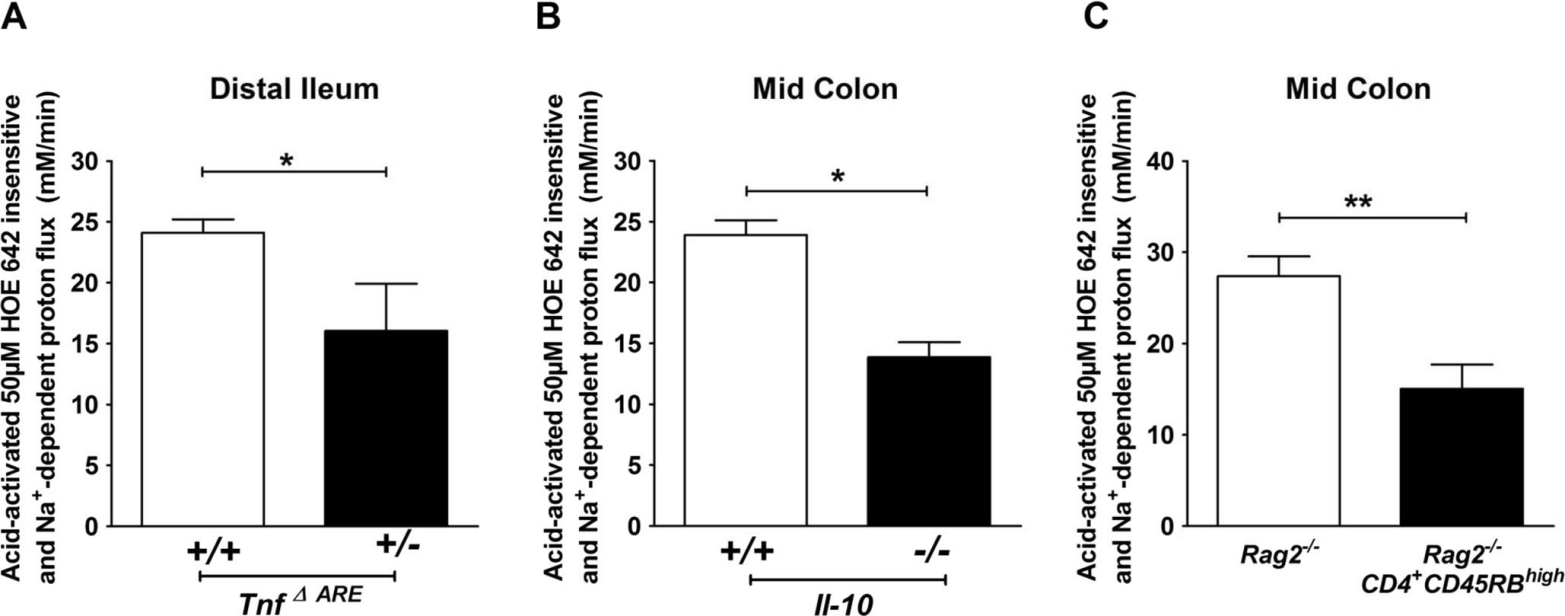
NHE3 activity measurements in the inflamed murine intestine. Acid-activated NHE3 activity was significantly decreased compared to the respective controls, **a** in the isolated ileal villi of *Tnf* ^ΔARE+/−^ mice, **b ** in isolated colonic crypts (10–15 crypts in each mice) from *Il-10* ^−/−^, and from **c** *Rag2* ^−/−^ CD4^+^ CD45^RBhigh^ mice. 20 μM of S1611 inhibited NHE3 activity even in *Tnf* ^ΔARE+/−^ and *Rag2* ^−/−^ CD4^+^ CD45^RBhigh^ mice, which shows that NHE3 activity was not completely lost in inflamed enterocytes (data not shown). A total of three to five pairs of inflamed and control mice were used for the experiments. *Bar graphs* are represented as mean ± SEM. *P < 0.05 and **P < 0.005

Acid-activated NHE3 transport rates were significantly lower in the enterocytes of microdissected *Tnf* ^ΔARE+/−^ ileal villi (Fig. 6a) as well as in the surface region of isolated colonic crypts of the 2 % DSS-induced *Il-10* ^−/−^ colon (Fig. 6b) and of the *Rag2* ^−/−^ CD4^+^ CD45^RBhigh^ colon (Fig. 6c).

### PDZK1 knockdown decreased acid-activated NHE3 activity in Caco-2BBe/hNHE3V cells

**Fig. 7:**
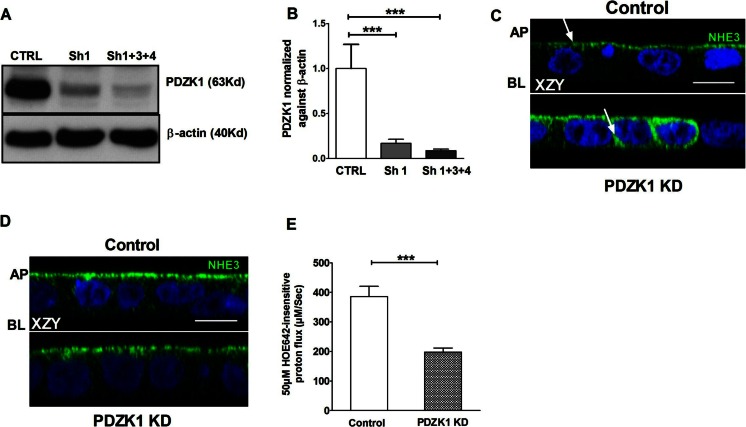
PDZK1 knockdown (PDZK1 KD) was established in Caco-2BBe/hNHE3V (C2N3) cells. PDZK1 KD cells display significantly reduced acid-activated NHE3 activity compared to control cells. **a** Total cell lysates from C2N3 cells infected with empty vector lentivirus (control/CTRL) and PDZK1 shRNAs (Sh1 and Sh1 + 3 + 4) were analyzed by Western blots. **b** Protein bands were quantified using image J software and the values were normalized against β-actin, *n* = 3. **c** Immunofluorescence images obtained by confocal microscopy in XZY plane showed a more diffused expression of NHE3-VSV-G in the cytoplasm of PDZK1 KD cells compared to control cells (*arrow* pointing to NHE3 staining); *AP* apical membrane, *BL* basolateral membrane. ***P < 0.0005. **d** Acid suicide was applied to improve the homogeneity and expression of NHE3 in both control and PDZK1 KD cells, after which a similar pattern of NHE3 expression was obtained in both control and PDZK1 KD cells. Those cells were then used for the NHE3 activity measurements. *Scale bar* represents 10 μM. **e** NHE3 activity was measured fluorometrically in C2N3/PDZK1 KD cells, which showed a significant reduction in NHE3 activity compared to control cells. *Bar graph* represents the results from three to five different passages and includes a total of at least nine cover slips (five regions of interest assessed in each cover slip) for each condition. *Bar graphs* are represented as mean ± SEM. ***P < 0.0005 compared to controls

A human NHE3 overexpressing colonic cell line Caco-2BBe/hNHE3V was established and PDZK1 expression was >70 % knocked down (Fig. 7a, b). Immediately after the PDZK1 knockdown, the cells displayed mislocalization of NHE3 to the intracellular and basolateral pool, and NHE3 expression was not present in all cells (Fig. 7c). To achieve more homogenous NHE3 expression levels, repeated cycles of “acid suicide selection” were performed both in the control and knockdown (KD) cells. When immunocytochemical analysis of the acid-selected cells revealed similar expression of NHE3 in the apical pole of the Caco-2BBe/hNHE3V empty vector control and the PDZK1 KD cells, they were used for NHE3 transport activity measurements (Fig. 7d). Despite robust apical NHE3 expression, the acid-activated NHE3 activity was significantly lower in PDZK1 KD cells than controls (Fig. 7e).

### NHE3 activity in *Pdzk1*^+/+^ and *Pdzk1*^+/−^ mice colonic enterocytes

**Fig. 8:**
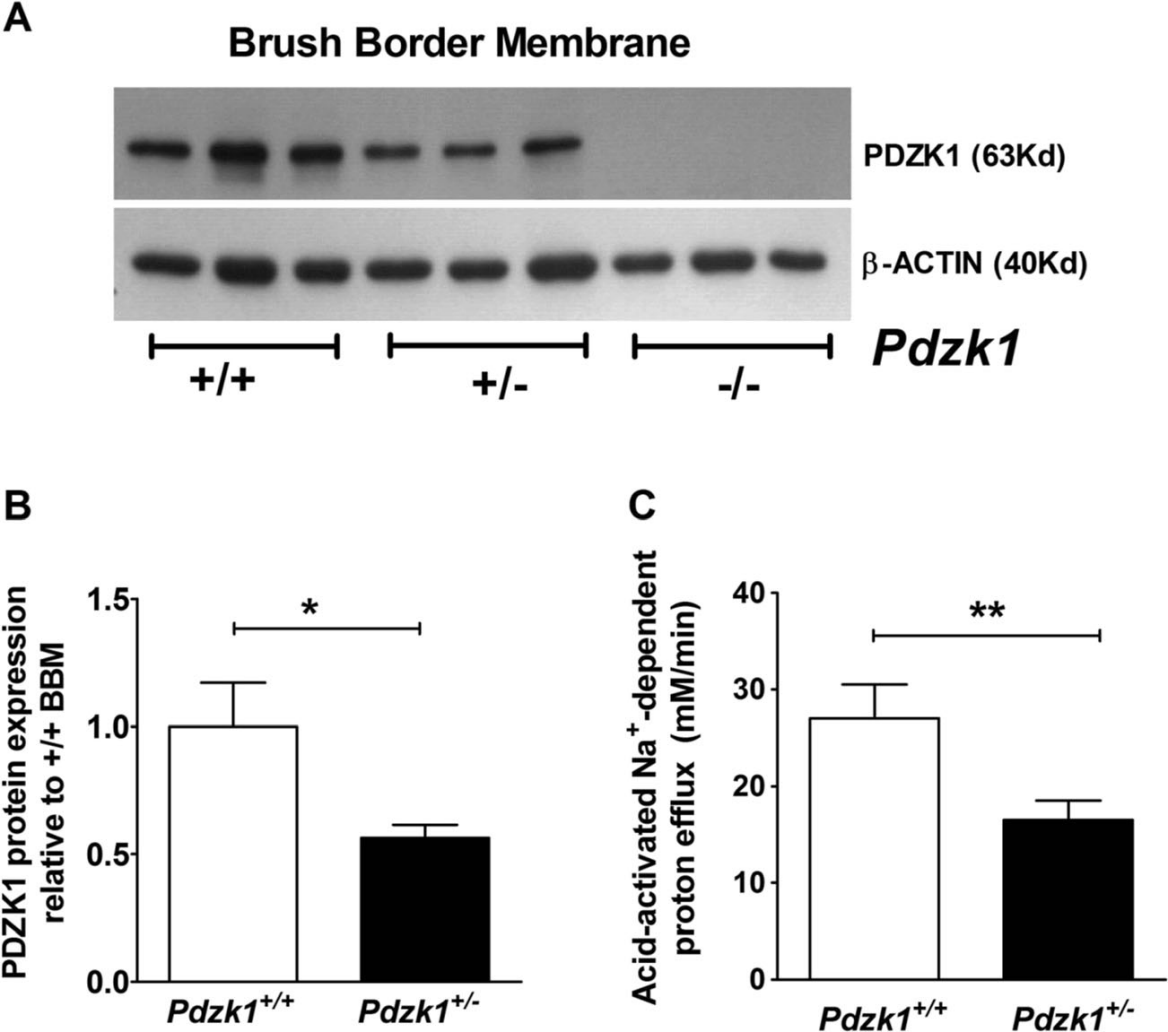
PDZK1 protein expression and NHE3 activity in *Pdzk1* ^+/−^ mice. **a** PDZK1 protein expression in the small intestinal BBM of *Pdzk1* ^+/+^, ^+/−^, and ^−/−^ mice was analyzed by Western blotting. **b** Protein bands were quantified using image J software and normalized against β-actin. PDZK1 protein abundance was more than 50 % reduced in the BBM from *Pdzk1* ^+/−^ compared to ^+/+^ mice. **c** Acid-activated NHE3 activity in cryptal mouth enterocytes from *Pdzk1* ^+/−^ colonic crypts was significantly lower than in colonocytes from control littermates. 6−8 crypts were measured from each colonic crypt preparations and averaged for each of the five pairs of mice. *Bar graphs* are represented as mean ± SEM. *P < 0.05, **P < 0.005, and ***P < 0.0005

Another approach to assess the consequence of reduced (but not absent) levels of PDZK1 expression on NHE3 activity was to study PDZK1 expression and NHE3 functional activity in enterocytes from PDZK1 heterozygotes. Acid-activated NHE3 activity was significantly decreased not only in the *Pdzk1* ^−/−^ colonocytes, as previously reported [9], but also in *Pdzk1* ^+/−^ compared to *Pdzk1* ^+/+^ colonocytes (Fig. 8c). This demonstrates that a reduction (but not absence) in PDZK1 protein expression, similar to that observed in inflamed colon, compromises colonocyte NHE3 function in a similar fashion to that observed in colonocytes from inflamed murine intestine.

## Discussion

The first part of the present project investigated whether the strong decrease in the expression of the PDZ-adaptor PDZK1 that we had previously observed in a mouse model for chronic colitis [24] was also found in humans with IBD. To do so, we obtained biopsies from the sigmoid colon of UC patients with inflammation, from UC patients in remission, and from controls with healthy mucosa, because we wanted to be able to correlate our findings with those of a previous study [40]. This previous study had demonstrated a functional defect in NHE3 regulation despite normal NHE3 expression and membrane localization, at least when studied with the methods that were available to us [40]. In the present study, we found PDZK1 mRNA expression to be strongly decreased in inflamed UC biopsies compared to noninflamed or control biopsies, whereas NHE3 and NHERF1 mRNA expression levels were not different from the controls (Fig. 1). NHERF2 mRNA was not investigated because its absence does not result in a decrease in acid-activated NHE3 activity in murine colon [8], and because of its low colonic expression [19]. In addition, the cross-reactivity of the NHERF2 antibody with the much more strongly expressed NHERF1 [35] would make interpretation of the results difficult. We also studied three inflammatory mouse models, each of which had an immunologically mediated intestinal inflammation, but with different segment predilection, acuity, and severity of inflammation. PDZK1 mRNA expression was significantly reduced in inflamed murine intestine, while NHERF1 was not (Fig. 2). NHE3 mRNA expression levels were either unaltered compared to noninflamed tissue or even increased, as in the *Rag2* ^−/−^ CD4^+^ CD45^RBhigh^ transfer colitis model. An increase in NHE3 mRNA expression during inflammation has also been observed in mild UC by Lohi et al. [26].

The cytokine mRNA expression levels in each mouse model showed a different pattern, with very high TNF-α as well IL-1β expression in the *Tnf* ^ΔARE+/−^ ileum, higher TNF-α than IL-1β expression in the *Rag2* ^−/−^ CD4^+^ CD45^RBhigh^ colon, and the *Il-10* ^−/−^ colon tissue showing most resemblance to that found in the UC biopsies. This difference in the cytokine expression pattern may in part be explained by the strong leukocyte infiltration in the *Tnf* ^ΔARE+/−^ [39] and the *Il-10* ^−/−^ [24] intestine, which was not observed to the same degree in the transfer colitis. An open question for future investigations remains whether the differences in the cytokine expression pattern observed in the different mouse models influence the degree of up- or downregulation of the investigated target genes.

We had previously published a preserved apical NHE3 location in the inflamed human colonocytes, despite a decrease in NHE3 functional activation by low pH_i_ [40]. In this study, NHE3 immunofluorescence was localized to the BBM in the inflamed enterocytes of all three mouse models. Given the importance of PDZK1 for NHE3 brush border membrane retention [19, 41], we were surprised by this finding. However, the *Pdzk1* ^−/−^ mouse also displays normal NHE3 localization in the BBM of the small and large intestine [7, 19]. This is associated with a severalfold increase in NHE3 mRNA expression, suggesting a problem with the stability of NHE3 membrane anchoring. It is possible that a similar situation exists in the inflamed enterocyte, which we cannot experimentally recognize, because the functional anatomy of the inflamed intestine is different from that in the normal intestine, with longer crypts/thicker villi and an altered distribution of NHE3 along the crypt/villus axis [2,40]. At the level of the enterocyte, however, the localization of NHE3 in the brush border membrane is correctly in the microvillar zone.

An incidental observation in the *Tnf* ^ΔARE+/−^ ileum was a stronger overlay between NHE3 and F-actin than in the noninflamed control (Fig. 4b). Also, the broadness of the F-actin zone in the microvilli was reduced in the *Tnf* ^ΔARE+/−^ ileum. Electron microscopical examination indeed revealed shorter microvilli in the apical zone of the *Tnf* ^ΔARE+/−^ villous enterocytes (Fig. 4). In view of these quite dramatic morphological changes in the inflamed BBM, it seems remarkable that so much NHE3 protein is nevertheless found in the BBM of the inflamed enterocytes.

We next assessed whether the NHE3 transport activity was also decreased in colonocytes or ileal enterocytes in the different mouse models (as had been observed to be the case in colonocytes from UC patients [40]). The Na^+^-dependent, HOE642-insensitive proton efflux rates after NH4^+^-induced acidification of surface colonic enterocytes and in the villous tip enterocytes in microdissected ileal villi, in the absence of CO_2_/HCO_3_^−^, are >80 % mediated by NHE3 [8, 9]. A significant decrease in enterocyte acid-activated NHE3 activity was observed in all three inflammation models (Fig. 6), as well as in the colonocytes in the cryptal mouth region of colonic crypts (where NHE3 is expressed strongly) isolated from biopsies from patients with active UC [40]. Fluid absorptive capacity was also decreased in the inflamed intestinal segments when compared to that of the same segments in noninflamed controls (Supplementary Figure 2). Thus, a decrease in PDZK1 expression was accompanied with a decreased acid-activated NHE3 activity in inflamed human and murine enterocytes.

The last, most important, and most difficult to address question of the study was that of the causality between the observed decrease in PDZK1 expression and the functional defect of NHE3. A complete lack of PDZK1 expression has been previously shown to result in a strong decrease in acid-activated NHE3 activity in colonocytes [9]. However, in the inflamed colon, PDZK1 expression levels were reduced but not absent. Since it is feasible that the multifunctional, multipartner PDZ adaptors of the NHERF family are expressed in levels that are not rate-limiting for the function of a given transporter in the enterocyte, we wondered how we can mimic the situation found in inflamed intestine. We searched for an intestinal cell line with endogenous PDZK1 expression, which was found to be the Caco-2BBe cell line. We established a Caco-2BBe cell line expressing the human NHE3 and performed lentiviral short hairpin RNA (shRNA)-mediated PDZK1 knockdown (KD). When similar NHE3 protein abundance was achieved in the brush border membrane, the acid-activated NHE3 activity was still significantly reduced in the PDZK1 KD cells (Fig. 7e), suggesting that PDZK1 plays a role in the functional regulation of NHE3 beyond mere membrane anchoring.

A second approach to study the effect of reduced, but not absent, PDZK1 expression was to measure the PDZK1 protein content in *Pdzk1* ^+/−^ mice. We found that in the small intestinal BBM of *Pdzk1* ^+/−^ mice, PDZK1 protein in the BBM was decreased to >50 % compared to WT (Fig. 8a, b). In colonic BBM, we could not accurately quantify PDZK1, because of actin band oversaturation when the large amount of protein necessary to be able to visualize the weak colonic PDZK1 band was loaded onto the gel, but the results seemed similar by eye. Therefore, we assessed acid-activated NHE3 activity in colonic crypts from *Pdzk1* ^+/−^ mice, and it was surprisingly strongly reduced compared to crypts from *Pdzk1* ^+/+^ littermates (Fig. 8c). Thus, both approaches to experimentally reduce enterocyte PDZK1 expression levels resulted in a weaker activation of NHE3 by low pH_i_, which cannot solely be explained by reduced NHE3 expression. This underlines the importance of PDZK1 for colonocyte NHE3 regulation and suggests that a strongly reduced PDZK1 expression in inflamed intestinal mucosa may be one of the reasons for colonic NHE3 dysfunction.

The molecular mechanisms of PDZK1-mediated enterocyte NHE3 regulation are largely unknown. In a study by Zachos et al., the authors used an adenoviral vector to acutely overexpress NHE3 in the respective control and PDZK1-knockdown Caco-2BBe cells [41]. They observed a decrease in NHE3 expression in the BBM, which was accompanied by a decrease in acid-activated NHE3 transport rate and a loss of carbachol-mediated inhibition of NHE3. This study confirmed our earlier observation in murine *Pdzk1* ^−/−^ intestine, in which both acid-activated enterocyte NHE3 activity was decreased, and forskolin- as well as carbachol-inhibition of NHE3 was abolished [9, 19]. However, in the *Pdzk1* ^−/−^ enterocytes of murine intestine, NHE3 protein expression in the BBM was not significantly decreased and NHE3 mRNA expression was severalfold upregulated, suggesting some compensatory mechanism [19]. Immediately after the PDZK1 knockdown, our Caco-2BBe/hNHE3 KD cells displayed a degree of mislocalization of NHE3 to the basolateral and intracellular pools (Fig. 7c). After several rounds of “acid suicide” selection, which allows cells that express Na^+^/H^+^ exchangers to survive prolonged incubation in acidic media, a fairly homogenous NHE3 distribution of similar intensity in the apical pole of both PDZK1 KD and control cells was observed (Fig. 7d). We did not measure NHE3 mRNA expression at the time, but we now know that the similar NHE3 abundance in the apical membrane of Caco-2BBe/hNHE3 KD and Caco-2BBe/hNHE3 cells was likely due to a selection of those KD cells with particularly high NHE3 mRNA expression (Luo and Giriprakash, unpublished data). Nevertheless, decreased acid-activated NHE3 activity was observed in the PDZK1 knockdown Caco-2BBe/hNHE3V cells compared to control cells. This suggests that low PDZK1 expression interferes with activation of NHE3 by a low pH_i_ even in the presence of sufficient apical NHE3 expression. We do not know much about the mechanisms of NHE3 activation by acid in the intestine, but studies in renal cells have outlined a complex signaling pathway involving both PYK2 as well as the ERK1/2 pathway as acid sensors, involving NHE3 exocytosis as well as possibly NHE3 phosphorylation (reviewed in [30]). PDZK1 has been identified as a major scaffolder in renal proximal tubule cells, interacting with not only a number of transporters but also a variety of kinases as well as with NHERF1 and NHERF2 [5, 14]. We therefore hypothesize that an inflammation-associated decrease in PDZK1 expression may weaken the kinase-anchoring cytoskeletal network (as described in [23, 27]) at the intestinal BBM. This may render not only NHE3 activation by acid less efficient, but also likely affects the regulation of a number of other intestinal transporters that interact with PDZK1. Further work in expression systems will be necessary to exactly define the role of PDZK1 in enterocyte brush border physiology and the cellular derangements that occur when PDZK1 is dysfunctional or missing.

An interesting and unexpected observation in the current study was the marked, previously unrecognized change of the microvillar structure in chronic intestinal inflammation, as observed in the inflamed *Tnf* ^ΔARE+/−^ ileum. Microvillar structural changes have been previously described in an acute inflammatory condition [31], but little information exists about chronic inflammation or about the underlying mechanisms of these changes. PDZK1 has been described to play a role in microvillus formation by interacting with NHERF1 and ezrin [21]. In order to investigate whether the change in microvillar structure in inflamed ileum may also be related to the strong downregulation of PDZK1, we investigated microvillar morphology in PDZK1 knockdown Caco-2BBe cells as well as in PDZK1-deficient mice by electron microscopy, but the length of the microvilli was not reduced in either situation (data not shown). Thus, the marked shortening of microvillar structure in the inflamed *Tnf* ^ΔARE+/−^ ileum is not related to the PDZK1 downregulation but to other cellular insults of chronic inflammation. This shortening of the microvilli may explain why the decrease in fluid absorption was so profound in *Tnf* ^ΔARE+/-^ ileum (Supplementary Figure 2), out of proportion of the decrease in acid-activated NHE3 activity (Fig. 6). Further work will be necessary to unravel the different molecular players in the inflammation-associated shortening of microvilli in chronic ileitis.

Could there be potential therapeutic consequences from our observations? We believe that recent research points to the fact that chronic intestinal inflammation does not just “destroy” the enterocytes, as was thought in the past. Brush border membrane transporter expression is surprisingly robust, but there seems to be a block on function. Normalization of ENaC-mediated sodium absorption was observed in distal colonic mucosal biopsies of Crohn’s disease patients after MAP kinase inhibition in vitro [42]. HCO_3_^−^ secretion was restored to normal in the colon of CFTR-deficient mice, which presented with increased inflammatory markers, after treatment with a peroxisome proliferator-activated receptor (PPAR)-γ agonist [16]. The few data that exist about PDZK1 promoter regulation suggest that PPAR-α activation increases PDZK1 promoter activity [36]. Thus, there may be a realistic chance that future study on PDZK1 gene regulation during intestinal inflammation may pave the way for therapeutic means to “disinhibit” intestinal absorption, even if the inflammation per se is not amenable to cure.

## Electronic supplementary material

Below is the link to the electronic supplementary material.

ESM 1 (DOCX 407 kb)
