# Supplementary material for: Evidence for a causal link between adaptor protein PDZK1 downregulation and Na+/H+ exchanger NHE3 dysfunction in human and murine colitis
Source: Pflugers Arch. 2014 Oct 2;467(8):1795–807. doi: 10.1007/s00424-014-1608-x (PMC4383727; doi:10.1007/s00424-014-1608-x)
Supplement: Supplementary file 1 — (DOCX 407 kb) [file 424_2014_1608_MOESM1_ESM.docx]

**SUPPLEMENTARY METHODS**

**RNA extraction and quantitative PCR protocol**

Total RNA was extracted from cells or different tissues using Qiagen RNeasy isolation kit (Qiagen, Hilden, Germany) according to the manufacturer’s protocol. RNA quality was assessed in Qiaxcel advanced system from Qiagen. 1.5µg of RNA was reverse transcribed using Superscript® III Reverse Transcriptase kit (Invitrogen, Darmstadt, Germany) according to the manufacturer’s protocol. cDNA was diluted to 1:20 and 5µl were used as a template for each PCR reaction, containing 12.5µl 2X MESA Green qPCR^Tm^ **(Eurogentec Deutschland GmbH**, Köln, Germany) master mix (including dNTPs and Taq Polymerase) and appropriate amount of primers up to a final amount of 25µl. For further details about PCR conditions and analysis see supplementary methods. For the gene expression study in human biopsies, we used the intestinal epithelial gene villin, as well as β-actin and Tbp (tatabox binding protein) as “housekeeping genes” and used a geometric mean of these values to normalize the target gene values, as described previously [29,40]. In mice, the intestinal epithelial gene villin was used as the control gene, after extensive pilot experiments had suggested that this is the best gene to take into account the crypt elongation in inflamed colon. Human primer sequences are given in supplementary table 3 and mouse primers were described previously [7,8,24,25,39,40].

**Generating *Rag2^−/−^*CD4^+^CD45RB^HIGH^ T cell transfer colitis model mice**

Specific pathogen-free BALB/c mice and recombination-activating gene (RAG)-2–deficient (*Rag2^−/−^*) mice on BALB/c background were bred in the animal facility of the Helmholtz Centre for Infection Research and were kept under specific pathogen-free conditions. All animal experiments were performed according to national and institutional guidelines. Mice were used at 8–12 wk of age.

**Antibodies**

The following mAbs were used for cell purification: FITC-conjugated anti–mouse CD45RB (clone 16A; PharMingen, California, USA) and APC–conjugated anti–mouse CD4 (clone RM4-5; PharMingen, California, USA).

**Cell isolation and fluorescent activated cell sorting**

Spleens were removed from BALB/c mice and single-cell suspensions were prepared by passing splenocytes through a 70 μm nylon mesh. Erythrocytes were removed from splenocytes by treatment with erythrocyte lysis buffer. Single cell suspensions were stained with APC–conjugated anti–mouse CD4 and FITC-conjugated anti–mouse CD45RB. Cell sorting was performed with a MoFlow cell sorter (DAKO Cytomation, Fort Collins, CO) to obtain CD4^+^CD45RB^high^ T cells. Cell population was >98% pure on reanalysis.

**T cell reconstitution and colitis development monitoring**

Rag2^−/−^ mice were injected intraperitoneally with 5×10^5^ sorted CD4^+^CD45RB^high^ T cells in PBS. The mice were then weighed daily and investigated for bloody anus and the development of pasty stools, which was evident several weeks after the injection. Colonic inflammation was validated retrospectively for each mouse by measurement of inflammatory cytokines in the mucosa.

**Acid suicide selection of Caco-22BBe/NHEV cells**

Caco-2BBe cells were transfected with full length NHE3 tagged with VSVG at C-terminus and stable cell lines were established using G418. These cells were grown at 37°C in a humidified atmosphere containing 5% CO_2_ and 95% O_2,_ in Dulbecco’s Modified Eagle Medium with 4.5g/.l^-1^ D‑glucose and sodium pyruvate (Life Technologies GmbH, Darmstadt, Germany) supplemented with 10% fetal calf serum (FCS), 100 units/ml penicillin, 100µg/ml streptomycin and 1% non‑essential amino acids. The cells were maintained with 800µg./ml^-1^ G418 antibiotic (Promo cell GmbH, Heidelberg, Germany) in the medium. Then the cells were subjected to acid selection: They were exposed to solution B and C for 1 hour each and then the cells were recovered by using solution A, which only contained 2mM Na^+^, for 1 hour (See supplementary table for solution compositions). This process was repeated until we achieved a strong BBM NHE3 expression.

**Lentiviral‑mediated PDZK1 knockdown in Caco‑2BBe/NHE3V Cells**

Bacterial glycerol stocks containing PLKO.1 lentiviral vector harboring five individual, small hairpin RNA (shRNA) constructs against human PDZK1 (SHCLNG-NM_002614) were obtained from Sigma (St.Louis, USA) and were used to generate lentiviral particles as described earlier [suppl. ref. 1]. To check the knockdown efficiency of individual shRNA against PDZK1 lentiviral transduction was done as follows:Caco-2BBe/NHE3V cells were plated at a density of 5×10^4^ cells per well in 24 well plates and incubated in 37° C and 5% CO_2_ until they reach 60-70% confluence. Then the medium from the wells was removed and replaced with medium containing 5µg/ml Polybrene. Equal volumes of lentivirus were added to the medium and cells were incubated overnight in Polybrene containing medium. Then the medium was replaced with normal medium and incubated for another 24 hours before continuing further. Caco‑2BBe/NHE3V cells were transduced with lentivirus harbouring individual shRNAs and 24 hours after transduction cells were harvested and checked for PDZK1 by Western blots. shRNA 1, 3 and 4 showed approximately 50 % inhibition. In order, to achieve higher knockdown of PDZK1, Caco‑2BBe/NHE3V cells were transduced with three shRNAs together, selected with 15 μg.ml^-1^ puromycin, and maintained with 10µg.ml^-1^ puromycin. PDZK1 protein expression was reduced to approximately 80% of control levels in the PDZK1 knockdown Caco‑2BBe cells (Figure 7A)

Western Analysis for PDZK1 in tissue lysates.

Protein concentration was estimated with a Bradford assay kit from Bio‑Rad (Munich, Germany) according to the manufacturer’s protocol. Total cellular proteins (50µg) were separated on 10% SDS poly acrylamide gel electrophoresis and transferred to polyvinylidene difluoride membranes (GE Healthcare Europe GmbH, Freiburg, Germany). Rabbit polyclonal antibodies against PDZK1 and β-actin were diluted in TBST (Tris buffered saline with 0.1% tween 20) containing 5% non-fat dry milk and blots were incubated overnight at 4°C, washed with TBST and incubated with secondary antibodies conjugated to horseradish peroxidise, washed with TBST and then developed using an enhancer chemiluminescence kit (GE Healthcare Europe GmbH, Freiburg, Germany). 50 µg of total protein was loaded on to the SDS-PAGE gel and probed with anti-PDZK1 antibody.

**
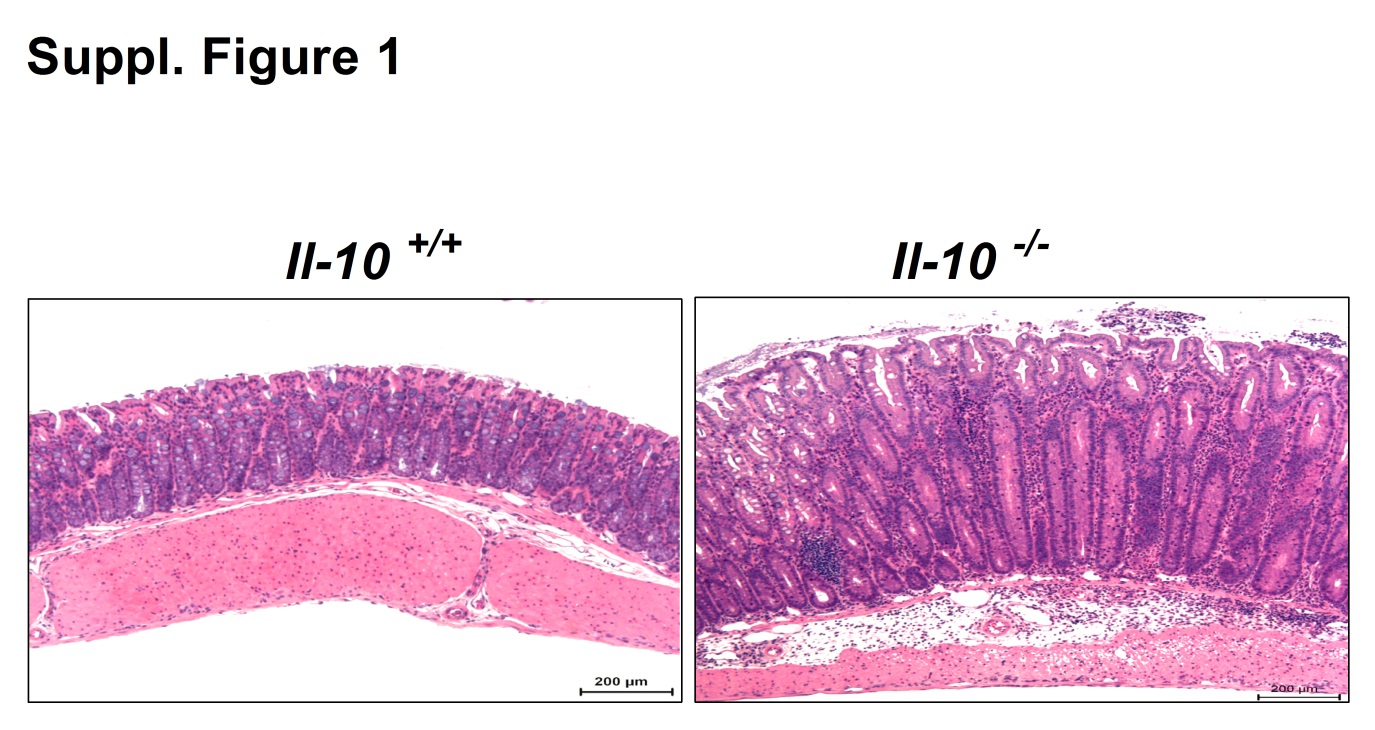
**

**Suppl. Figure 1. Morphological changes and cytokine expression in inflamed mice intestine.**

H&E staining of formalin fixed mid-distal colon of *IL-10^+/+^* (Left panel) and *IL-10**^-/-^* (right panel). Epithelial hyperplasia and an increased amount of lamina propria lymphocytes were observed in *IL-10^-/-^* colon compared to their WT controls. Scale bar represents 200µm.

**
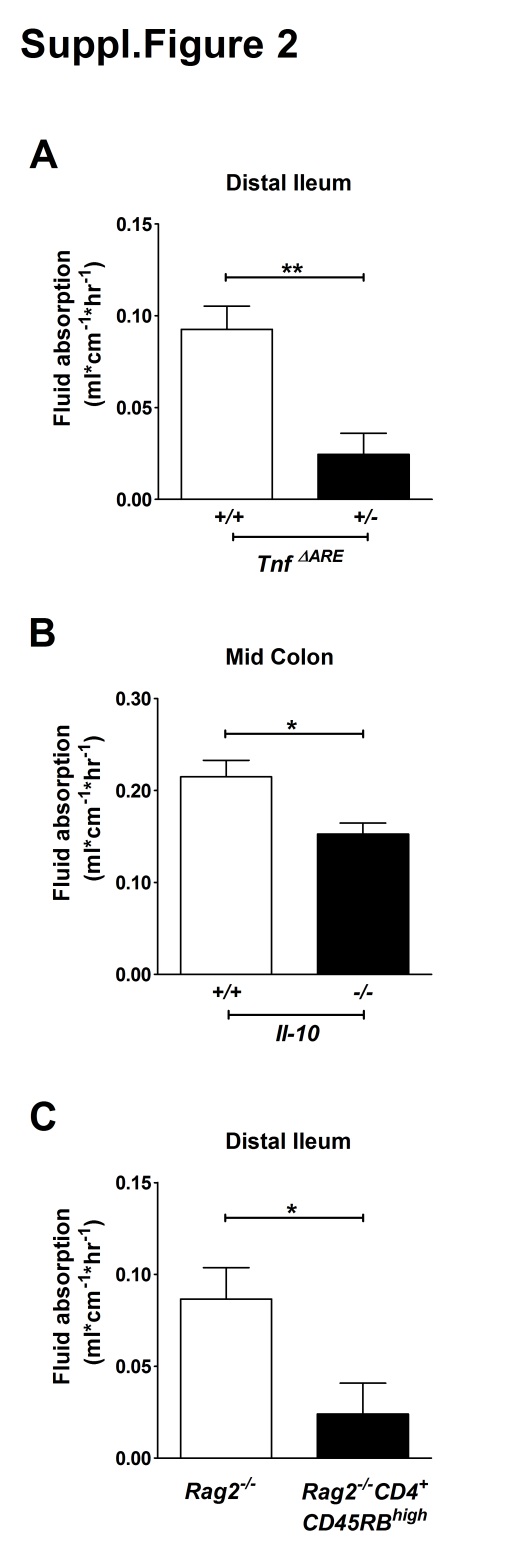
**

**Suppl. Figure 2. Fluid absorption in the inflamed mice intestine.**

Fluid absorption was decreased in A) *TNF^ΔARE+/-^* mice compared to their controls by more than 60%, B) in the proximal‑mid colon of *IL‑10^‑/‑^* mice compared to their controls by 30% and C) in the distal ileum of *Rag 2-/-* CD^4+^CD^45^RB^high^ colitis mice, compared to its controls was by more than 60%. It is obvious that while fluid absorption is decreased in all inflamed intestinal segments compared to the respective segments in noninflamed mice, the degree of reduction in fluid absorption does not correlate closely with the degree in reduction of acid-activated NHE3 activity. This may have many reasons, but one potential reason for the relatively mild reduction of fluid absorption in the IL-10^-/-^ may be the marked crypt elongation (suppl. Fig. 1) with an increased NHE3 expression zone within the crypts [suppl ref. 2]. The marked decrease in fluid absorptive rate in the ileum of the *TNF^ΔARE+/-^* mice may be due to the short microvilli, in addition to a decreased nah exchange rate, and other reasons. Data is represented as mean ± SEM. * *P*< .05and ** *P* < .005. n= 5 mice pairs.

**Supplementary Table 1: Healthy control Patient details**

| **Patient nr.** | **Age** | **Sex** | **Reason for colonoscopy** | **Degree of inflammation** |
| --- | --- | --- | --- | --- |
| 1 | 58 | F | Hemorrhoids control check | No Inflammation |
| 2 | 51 | F | Hemorrhoids control Check | -do- |
| 3 | 58 | F | Acute abdominal pain with no blood and diarrhea | -do- |
| 4 | 28 | M | Obscure bleeding, rheumatism | -do- |
| 5 | 38 | M | Liver cirrhosis patient, clarification for transplantation | -do- |
| 6 | 73 | F | Unexplained upper abdominal discomfort and weight loss (**+)** | -do- |
| 7 | 50 | F | C2 liver cirrhosis and renal failure | -do- |
| 8 | 65 | F | Obscure bleeding for 5 days with out diarrhea | -do- |
| 9 | 37 | M | Fainting | -do- |
| 10 | 51 | M | Diverticulosis | -do- |
| 11 | 41 | F | Cancer screening | -do- |
| 12 | 60 | F | Anemia and tarry stool (four weeks before) | -do- |
| 13 | 27 | F | Stool frequency 1-3 times per day, no blood, pappy, mild abdominal pain, skin abscesses (§) | -do- |
| 14 | 30 | F | Abdominal pain | -do- |
| 15 | 57 | F | Cancer screening | -do- |
| 16 | 52 | F | Polypectomy | -do- |

-do- means no inflammation

**Supplementary Table 2: Ulcerative colitis patients details**

| **Patient nr.** | **Age** | **Sex** | **Medication** | **Degree of inflammation** |
| --- | --- | --- | --- | --- |
| 1 | 35 | Female | Sulfasalazin | non inflamed |
| 2 | 59 | Male | Corticosteroid,  Mycophenolat-Mofetil | non inflamed |
| 3 | 43 | Male | 5-ASA | non inflamed |
| 4 | 37 | Male | 5-ASA, Corticosteroid, Heilschlamm | non inflamed |
| 5 | 31 | Male | 5-ASA | non inflamed |
| 6 | 39 | Male | 5-ASA | non inflamed |
| 7 | 42 | Male | 5-ASA | non inflamed |
| 8 | 62 | Male | - | non inflamed |
| 9 | 40 | Male | 5-ASA | non inflamed |
| 10 | 74 | Male | 5-ASA | non inflamed |
| 11 | 43 | Female | 5-ASA | non inflamed |
| 12 | 53 | Male | 5-ASA | non inflamed |
| 13 | 38 | Male | 5-ASA, Corticosteroid, Tacrolimus | non inflamed |
| 14 | 39 | Female | Sulfasalazin, Corticosteroid | Mild |
| 15 | 62 | Female | 5-ASA | Mild |
| 16 | 38 | Female | 5-ASA, Infliximab | Mild |
| 17 | 62 | Male | 5-ASA, Lecithin | Mild |
| 18 | 26 | Male | 5-ASA, Golimumab, Lecithin | Mild |
| 19 | 20 | Male | 5-ASA | Mild |
| 20 | 28 | Male | 5-ASA | Mild |
| 21 | 49 | Male | 5-ASA, Corticosteroid | Mild |
| 22 | 30 | Male | 5-ASA, Corticosteroid, Infliximab | Moderate |
| 23 | 48 | Male | 5-ASA | Moderate |
| 24 | 19 | Male | 5-ASA, Mycophenolat-Mofetil, Corticosteroid, Tacrolimus | Moderate |
| 25 | 19 | Male | 5-ASA, Mycophenolat-Mofetil, Corticosteroid, Tacrolimus | Moderate |
| 26 | 32 | Male | Sulfasalazin, Golimumab | Severe |
| 27 | 23 | Male | 5-ASA | Severe |

**Supplementary Table 3: PCR Primers details**

Human Specific PCR primers

| **Name** | **Sequence** |
| --- | --- |
| **NHE3** forward | 5´-AGAAGCGGAGAAACAGCAG-3´ |
| **NHE3** reverse | 5´-TGGTGACACTAGCCAGGAAC-3´ |
| **NHERF1** forward | 5´-TGGACAGGGAAACTGACGAG-3´ |
| **NHERF1** reverse | 5´-GACTGTTCTCCTTCTGTATCTCC-3´ |
| **PDZK1** forward | 5´-TCCCTATTGTTTCCTCCCTG-3´ |
| **PDZK1** reverse | 5´-GGAAGAAGAATGTGAGGCTG-3´ |
| **TNF-α** forward | 5´-AGGGACCTCTCTCTAATCAGC-3´ |
| **TNF-α** reverse | 5´-TCAGCTTGAGGGTTTGCTAC-3´ |
| **IL-1β** forward | 5´-AATTTGAGTCTGCCCAGTTCCCC-3´ |
| **IL-1β** reverse | 5´-AGTCAGTTATATCCTGGCCGCC-3´ |
| **IFN-γ** forward | 5´-CCAACGCAAAGCAATACATG-3´ |
| **IFN-γ** reverse | 5´-TTTTCGCTTCCCTGTTTTAGC-3´ |
| **Villin** forward | 5´-CTATGCCAACACCAAGAGAC-3´ |
| **Villin** reverse | 5´-CCCAGACATCTAGTAGGAACAC-3´ |
| **β-Actin** forward | 5´-CTGGCACCCAGCACAATG-3´ |
| **β-Actin** reverse | 5´-CCGATCCACACGGAGTACTT-3´ |
| **TBP** forward | 5´-CACGAACCACGGCACTGATT-3´ |
| **TBP** reverse | 5´-TTTTCTTGCTGCCAGTCTGG-3´ |

**Supplementary Table 4: Solutions for pH Fluorimetry and acid selection.**

| **Solution** | **Na^+^**  **(mM)** | **Cl^-^**  **(mM)** | **K^+^**  **(mM)** | **HPO4^-2^**  **(mM)** | **Mg^2+^**  **(mM)** | **Ca^2+^**  **(mM)** | **NH4^+^**  **(mM)** | **TMA^+^**  **(mM)** | **HEPES**  **(mM)** | **Glucose**  **(mM)** |
| --- | --- | --- | --- | --- | --- | --- | --- | --- | --- | --- |
| **A** | 130 | 141 | 6 | 0.5 | 1 | 2 | -- | -- | 11 | 25 |
| **B** | -- | 141 | 6 | 0.5 | 1 | 2 | 32 | 98 | 11 | 25 |
| **C** | -- | 141 | 6 | 0.5 | 1 | 2 | -- | 130 | 11 | 25 |
| **D** | -- | 141 | 130 | 0.5 | 1 | 2 | -- | -- | 11 | 25 |

**SUPPLEMENTARY REFERENCES**

1 He P, Klein J, Yun CC. Activation of Na+/H+ exchanger NHE3 by angiotensin II is mediated by inositol 1,4,5-triphosphate (IP3) receptor-binding protein released with IP3 (IRBIT) and Ca2+/calmodulin-dependent protein kinase II. *J Biol Chem* 2010;**285**:27869-78.

2. [Seidler U](file:///C:\pubmed%3fterm=Seidler%20U%5bAuthor%5d&cauthor=true&cauthor_uid=17057206)^1^, [Lenzen H](file:///C:\pubmed%3fterm=Lenzen%20H%5bAuthor%5d&cauthor=true&cauthor_uid=17057206), [Cinar A](file:///C:\pubmed%3fterm=Cinar%20A%5bAuthor%5d&cauthor=true&cauthor_uid=17057206), [Tessema T](file:///C:\pubmed%3fterm=Tessema%20T%5bAuthor%5d&cauthor=true&cauthor_uid=17057206), [Bleich A](file:///C:\pubmed%3fterm=Bleich%20A%5bAuthor%5d&cauthor=true&cauthor_uid=17057206), [Riederer B](file:///C:\pubmed%3fterm=Riederer%20B%5bAuthor%5d&cauthor=true&cauthor_uid=17057206). Molecular mechanisms of disturbed electrolyte transport in intestinal inflammation. NYAS 2006; 1072:262-75.
